# Refugee Integration Outcomes cohort study: Evidence for policy and planning

**DOI:** 10.23889/ijpds.v8i2.2198

**Published:** 2023-09-14

**Authors:** Sam Frowen, Nicky Rogers, Alan Evans, Hannah Toft, Angela Black, Tilly Skipper, Caroline Peate, Raoul Hodgson

**Affiliations:** 1 Office For National Statistics, Newport, United Kingdom; 2 Office For National Statistics, Titchfield, United Kingdom; 3 Home Office, London, United Kingdom

Evidence on refugee integration outcomes in the UK is lacking, partly due to an absence of datasets which permit refugees to be identified. The RIO longitudinal cohort study designed in collaboration with the Home Office aims to address this by linking administrative data longitudinally.

RIO covers cohorts granted asylum and refugees resettled in England & Wales via the Vulnerable Persons and Vulnerable Children’s resettlement Schemes between 2015 and 2020. Linked data include the Personal Demographics Service from the NHS and Exit Checks from the Home Office. Census 2021 data have also been linked to the study. Deterministic linkage algorithms addressed different naming conventions across a wide set of cultures and administrative data quality. Associative linkage methods were developed to match residuals from the deterministic stage to their corresponding household if present. We conducted our own internal quality analysis to assess the quality of our linkage algorithms to improve our methodology ahead of incorporating new administrative data such as HMRC and DWP data.

Experimental analysis has looked at social and economic outcomes for these refugee cohorts. Linkage to NHS data helps us understand access to health services and time taken to access these services once resettled. Census 2021 data provide a rich understanding of integration outcomes up to 6 years after arrival or grant of asylum. We demonstrate the potential of linked census data to provide evidence on housing, education, health, access to the labour market, education, households and secondary migration but also how this varies for asylum and refugee cohorts but also by age, sex and geographical region.

RIO is aligned to the ONS strategic objective of inclusivity and recommendations made by the UK National Statistician’s Inclusive Data Task Force. RIO will ultimately help inform local authorities, government, charities and other organisations with resource allocation for these vulnerable populations. We are planning to make this dataset available to Accredited Researchers via the ONS Secure Research Service and the Integrated Data Service (IDS).

